# Biological rational for sequential targeting of Bruton tyrosine kinase and Bcl-2 to overcome CD40-induced ABT-199 resistance in mantle cell lymphoma

**DOI:** 10.18632/oncotarget.3275

**Published:** 2015-03-05

**Authors:** David Chiron, Christelle Dousset, Carole Brosseau, Cyrille Touzeau, Sophie Maïga, Philippe Moreau, Catherine Pellat-Deceunynck, Steven Le Gouill, Martine Amiot

**Affiliations:** ^1^ INSERM, UMR892 - CNRS, UMR 6299, Université de Nantes, France; ^2^ Service d’Hématologie Clinique, Unité d’Investigation Clinique, Centre Hospitalier Universitaire de Nantes, France; ^3^ CIC, INSERM, Nantes, France

**Keywords:** ABT-199, mantle cell lymphoma, apoptosis, Bcl-2 family members, ibrutinib

## Abstract

The aggressive biological behavior of mantle cell lymphoma (MCL) and its short response to current treatment highlight a great need for better rational therapy. Herein, we investigate the ability of ABT-199, the Bcl-2-selective BH3 mimetic, to kill MCL cells. Among MCL cell lines tested (*n* = 8), only three were sensitive (LD_50_ < 200 nM). In contrast, all primary MCL samples tested (*n* = 11) were highly sensitive to ABT-199 (LD_50_ < 10 nM). Mcl-1 and Bcl-x_L_ both confer resistance to ABT-199-specific killing and *BCL2/(BCLXL + MCL1)* mRNA ratio is a strong predictor of sensitivity. By mimicking the microenvironment through CD40 stimulation, we show that ABT-199 sensitivity is impaired through activation of NF-kB pathway and Bcl-x_L_ up-regulation. We further demonstrate that resistance is rapidly lost when MCL cells detach from CD40L-expressing fibroblasts. It has been reported that ibrutinib induces lymphocytosis *in vivo* holding off malignant cells from their protective microenvironment. We show here for two patients undergoing ibrutinib therapy that mobilized MCL cells are highly sensitive to ABT-199. These results provide evidence that *in situ* ABT-199 resistance can be overcome when MCL cells escape from the lymph nodes. Altogether, our data support the clinical application of ABT-199 therapy both as a single agent and in sequential combination with BTK inhibitors.

## INTRODUCTION

Mantle cell lymphoma (MCL) is a mature B-cell neoplasm characterized by an aggressive clinical course. Although recent progress in the treatment of MCL patients has yielded high complete response rates, relapse invariably occurs and MCL remains incurable [1, 2]. This highlights the necessity to uncover the mechanisms involved in tumor cell survival and drug resistance. The primary oncogenic event in MCL is the t(11;14) translocation resulting in cyclin D1 overexpression and consequent cell cycle dysregulation [3]. However, additional mechanisms are required for MCL progression, including alteration of the mitochondrial apoptotic pathway, which mainly determines cell fate [4–6]. Among the critical components of the apoptotic machinery, the anti-apoptotic protein Bcl-2 is overexpressed in MCL cells and may then represent an attractive target for innovative therapy. BH3 mimetics, such as ABT-737 and ABT-263 (navitoclax), which bind with high affinity Bcl-2 and Bcl-x_L_ have been developed. They displace pro-apoptotic proteins from Bcl-2 and Bcl-x_L_ and induce apoptosis through a Bax or Bak dependent manner [7, 8]. Recently, navitoclax has demonstrated antitumor activity in B-cell malignancies but its clinical development was limited by the significant thrombocytopenia caused by Bcl-x_L_ inhibition [9]. To overcome this toxicity, the first-in-class orally bioavailable Bcl-2-selective BH3 mimetic ABT-199 was developed and thus far has shown very promising antitumor activity while sparing platelets in chronic lymphoid leukemia (CLL) and B-cell lymphomas, including MCL [10, 11].

Recently it has been demonstrated that tumor microenvironment strongly influence proliferation, survival and drug resistance in MCL cells [12, 13]. Moreover, microenvironment has been implicated in BH3 mimetic resistance via the modulation of Bcl-2 family proteins expression in CLL cells [14–16]. In the present study, we investigated the apoptotic efficiency of ABT-199 in both MCL cell lines and primary cells by integrating the key role of the microenvironment. This led us to propose a rational combination strategy to overcome microenvironment-dependent ABT-199 resistance through prior induction of cellular egress in peripheral blood using the selective BTK inhibitor ibrutinib [17].

## RESULTS

### Mantle cell lymphoma sensitivity to ABT-199 correlates with *BCL2/(MCL1+BCLXL)* gene expression ratio

To determine sensitivity of MCL cells to ABT-199, cell lines (*n* = 8) were treated with increasing doses of ABT-199 for 48 hours. As shown in Table 1A, the efficacy of ABT-199 was heterogeneous among MCL cell lines. Indeed, MAVER-1, MINO and GRANTA-519 cells were found to be highly sensitive to ABT-199 (LD_50_ from 15 to 200 nM) while Z138, JeKo-1, REC-1, JVM2 and UPN-1 were found to be resistant (LD_50_ from 1000 to 10000 nM) (Table 1A). We next addressed ABT-199 sensitivity in primary MCL cells obtained from peripheral blood of 11 patients at diagnosis or relapse. In contrast to MCL cell lines, low doses of ABT-199 (10 nM) induced cell death in all samples, ranging from 53% to 98% indicating that primary cells presented a LD_50_ < 10 nM (Table 1B).

**Table 1:** MCL cell sensitivity to ABT-199 correlates with the *BCL2/(MCL1+BCLXL)* ratio **(A)** Cell lines were cultured with increasing doses of ABT-199 for 48 hours to determine the median lethal dose (LD_50_: 15-10000 nM). **(B)** MCL cells from peripheral blood were obtained after gradient density centrifugation on Ficoll Hypaque. MCL cells were cultured with 10 nM of ABT-199 for 24 hours. Diag: diagnosis, Rel: relapse, ND: data not determined. The relative expression of *BCL2*, *MCL1 and BCLXL* mRNA was defined on purified CD19^+^ cells as described in the Methods section and *BCL2/(MCL1+BCLXL)* mRNA ratio is indicated. Analysis of *BCLXL*, *MCL1 and BCL2* relative expression in primary MCL cells and cell lines are shown in Figure 1A. Correlation between *BCL2/(MCL1+BCLXL)* ratio and ABT-199 sensitivity is shown in Figure 1B.

A					
Cell lines	LD_50_ ABT-199	*BCL2*	*MCL1*	*BCLXL*	*(BCL2)/(MCL1+BCLXL)*
**MAVER-1**	15	6.55	0.92	2.01	**2.24**
**MINO**	100	1.38	0.60	1.25	**0.75**
**GRANTA-519**	200	3.90	0.89	2.16	**1.28**
Z138	1000	0.98	0.67	1.95	0.37
REC-1	5000	0.68	1.79	3.77	0.12
JVM2	5000	1.48	1.44	2.83	0.35
JeKo-1	7000	1.00	1.00	1.00	0.50
UPN-1	10000	0.01	0.45	1.10	0.00

B						
Patient	Status	ABT-199 (10 nM)% of cytotoxicity	*BCL2*	*MCL1*	*BCLXL*	*(BCL2)/(MCL1+BCLXL)*
**#1**	Diag	53	2.76	0.82	0.27	**2.53**
**#2**	Diag	56	2.10	0.78	1.07	**1.14**
**#3**	Rel	56	3.77	0.9	1.1	**1.96**
**#4**	Rel	58	ND	ND	ND	ND
**#5**	Diag	62	3.57	1.59	0.95	**1.41**
**#6**	Diag	62	1.69	1.08	0.32	**1.22**
**#7**	Rel	66	1.78	0.8	0.5	**1.43**
**#8**	Diag	69	1.20	0.90	0.30	**1.00**
**#9**	Diag	69	2.05	1.03	1.11	**0.96**
**#10**	Diag	77	1.51	0.40	0.30	**2.16**
**#11**	Rel	98	ND	ND	ND	ND

**Figure 1:**
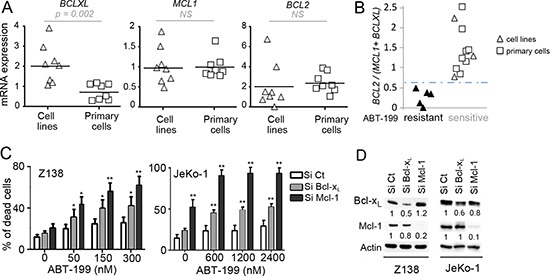
Influence of Bcl-2 family anti-apoptotic proteins on ABT-199 sensitivity in MCL cells **(A)** Analysis of the relative expression of *BCLXL, MCL1 and BCL2* mRNA by RT-qPCR in MCL cell lines (*n* = 8) and primary MCL cells (*n* = 8). The relative expression was normalized to JeKo-1 cell line. **(B)** The *BCL2/(MCL1+BCLXL)* mRNA ratio correlates with ABT-199 sensitivity in MCL cells. Cells with a LD_50_ < 200 nM were defined as sensitive whereas cells with a LD_50_ > 1000 nM were defined as resistant. The cut-off value (0.67) was determined as the mean of *BCL2/(MCL1+BCLXL)* ratio of resistant cells + (standard deviation) x 2 (True positive rate: 100%) **(C)** Both Mcl-1 and Bcl-x_L_ confer primary resistance to ABT-199. Z138 and JeKo-1 cell lines were transfected with Si Control (Ct), Mcl-1 or Bcl-x_L_. Following transfection, cells were treated with ABT-199 for 24 hours and cell death was quantified by Apo2.7 staining. *p*-value was determined using the paired Student's *t* test: *p < .05; **p < .01. **(D)** The protein levels of Mcl-1 and Bcl-x_L_ were determined by immunoblotting.

We next analyzed the sensitivity to ABT-199 in regards to the expression of anti-apoptotic Bcl-2 family members determined by RT-qPCR in both cell lines and primary samples (Table 1). Whereas *BCL2* and *MCL1* levels were similar in both cell lines and primary cells, *BCLXL* mRNA expression was significantly lower in primary MCL cells (*p* = 0.002) (Fig. 1A). We previously reported that the *BCL2/MCL1* ratio was a powerful biomarker for predicting ABT-737 sensitivity in MCL [18]. Using both MCL cell lines and primary cells we found here a direct correlation between ABT-199 sensitivity threshold and *MCL1* and *BCLXL* anti-apoptotic gene expression. Indeed, whereas neither *BCL2/MCL1 nor BCL2/BCL2XL* mRNA ratios were sufficient (Supplementary Fig. S1A), *BCL2/(MCL1+BCLXL)* mRNA ratio discriminated sensitive from resistant MCL cells with a cut-off value of 0.67 (*p* < 0.001; Fig. 1B). Of note, the Bcl-2/(Mcl-1+Bcl-x_L_) protein ratio strongly correlated with the mRNA ratio in MCL cells (*p* < 0.001; Supplementary Fig. S1B–S1C). Taken together, these data suggest that both Bcl-x_L_ and Mcl-1 expression play a role in ABT-199 resistance in MCL through increase of the apoptotic threshold.

To investigate the role of Bcl-x_L_ and Mcl-1 in ABT-199 response, these anti-apoptotic proteins were knocked down using siRNA in both Z138 and JeKo-1 resistant cells. Mcl-1 silencing sensitized both cell lines to lower doses of ABT-199 confirming the critical role of Mcl-1 in BH3-mimetics resistance as previously shown (Fig. 1C) [18]. Bcl-x_L_ silencing also sensitized Z138 and JeKo-1 cells to ABT-199, to a lesser extent than Mcl-1 silencing which may be explained by a lower silencing efficacy (Fig. 1C–1D). These results confirm that both Bcl-x_L_ and Mcl-1 determine ABT-199-specific response in MCL cells.

### CD40 stimulation reduces ABT-199 efficacy in MCL cells

**Figure 2:**
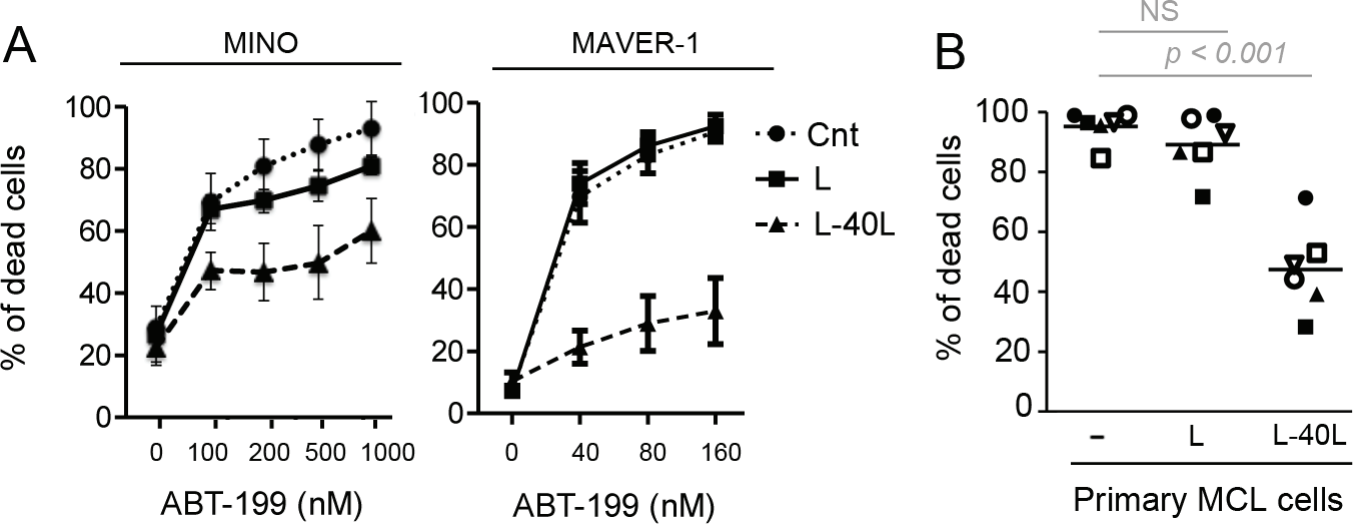
CD40 stimulation resulted in strong resistance to ABT-199 **(A)** MCL cell lines were cultured alone or on either parental fibroblast L or CD40L-expressing fibroblasts L (L-40L) for 24 hours before being exposed to ABT-199. Cell death was assessed in triplicate by using Apo-2.7 staining. **(B)** Primary MCL cells were cultured alone or on either L or L-40L cells for 24 hours then exposed to 25 nM ABT-199 for 48 hours. Apoptosis was determined by Apo2.7 and CD19 staining.

Because MCL cells mainly reside in lymph nodes we next asked whether microenvironment interactions could impact their sensitivity to ABT-199. In order to mimic the lymph node microenvironment where CD40-CD40L interaction takes place, ABT-199 sensitive MCL cell lines (MINO and MAVER-1) were cultured on CD40L-expressing fibroblast L cells (L-40L). Co-culture with L-40L dramatically reduces their sensitivity to ABT-199 while co-culture with parental fibroblast L cells failed to induce significant resistance (Fig. 2A). Of note, primary MCL cells from patients were also significantly more resistant to ABT-199 when cultured on L-40L with 25 nM of ABT-199 (*n* = 6; *p* < 0.001) (Fig. 2B). By contrast, culture of MINO cells with conditioned medium from L-40L culture or with bone marrow stromal cells (HS5) failed to reduce ABT-199 sensitivity (data not shown). These results indicate that the CD40 pathway is directly involved in the resistance to ABT-199 in both MCL cell lines and primary cells.

### Bcl-x_L_ up-regulation by CD40 stimulation confers resistance to ABT-199

**Figure 3:**
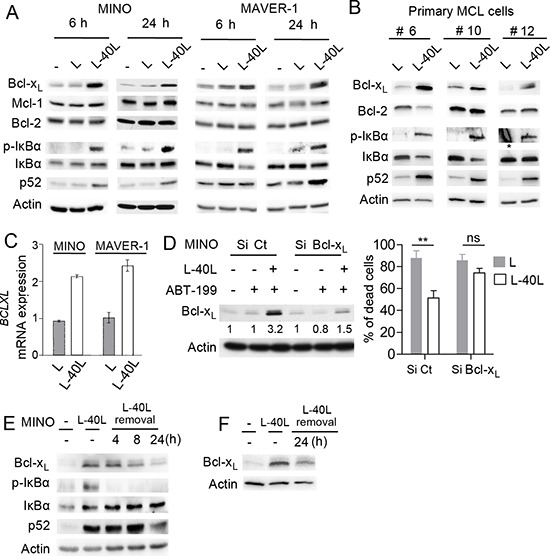
Up-regulation of Bcl-xL by CD40 stimulation confers ABT-199 resistance to MINO cells **(A)** Immunoblot analysis of Bcl-2 family (Bcl-x_L_, Mcl-1, Bcl-2) and NF-kB (pIkB, IkB, p52) proteins expression in MINO and MAVER-1 cells cultured on either parental L or L-40L for 6 and 24 hours. **(B)** Immunoblot analysis of indicated proteins in primary MCL samples cultured on either L or L-40L cells for 24 hours. * indicates non-specific staining **(C)** *BCLXL* mRNA was measured by RT-qPCR in MINO and MAVER-1 cells treated as indicated in A. **(D)** Left panel: MINO cells were transfected with Si Control (Ct) or Bcl-x_L_ and silencing at the protein level was confirmed by immunoblotting. Right panel: following 24 hours of transfection, cells were cultured on either L or L-40L cells before being treated with ABT-199 for additional 48 hours. **(E-F)** MINO cells (E) and primary MCL cells (F) cultured on L-40L for 24 hours were removed from coculture support and cultured alone for indicated time and analyzed by immunoblotting.

To assess the involvement of Bcl-2 family proteins in ABT-199 resistance under CD40 stimulation, we examined their expression following co-culture with either L or L-40L fibroblasts. CD40 stimulation of ABT-199 sensitive MCL cell lines resulted in a strong up-regulation of Bcl-x_L_ proteins within 6 to 24 hours of co-culture while no modification of Mcl-1 or Bcl-2 levels was observed (Fig. 3A). The CD40-induced Bcl-x_L_ expression was then confirmed at the mRNA level in MINO and MAVER-1 cell lines (Fig. 3C).

CD40 activation is known to potently activate both classical and alternative NF-kB pathway [19], which mediate Bcl-x_L_ upregulation [20]. Thus, we investigated NF-kB activation through the phosphorylation of IκBα and accumulation of p52 in MCL cell lines under CD40 stimulation. As shown in Fig. 3A both classical (p-IkB) and alternative (p52) NF-kB pathways were activated under CD40 stimulation in both MINO and MAVER-1 cells (Fig. 3A). Of interest, CD40 stimulation (24 hours) of primary MCL cells (*n* = 3) also demonstrated a strong Bcl-x_L_ upregulation associated to the activation of both classical and alternative NF-kB pathways (Fig. 3B).

To further confirm the critical role of Bcl-x_L_ in the resistance to ABT-199 induced by CD40 stimulation, we transiently silenced its expression by RNAi. siRNAs against *BCLXL* in MINO cells impaired protein up-regulation upon CD40 stimulation and significantly prevented the resistance to ABT-199 confirming a critical role for Bcl-x_L_ in cell protection (Fig. 3D).

Because MCL cells frequently disseminate from lymph nodes into circulation, we mimicked this process by removing MINO or primary MCL cells from L40-L before assessing Bcl-x_L_ levels in order to determine whether resistance to ABT-199 would persist after detachment from L40-L. We observed a rapid decrease of IkBα phosphorylation as well as a progressive reduction of p52 and Bcl-x_L_ protein level to baseline levels within 24 hours after detachment from L40-L (Fig. 3E–3F). Taken together, these results suggest that ABT-199 resistance due to CD40/CD40L interaction is linked to Bcl-x_L_ upregulation and that sensitivity could be rapidly restored after egress of MCL cells from their protective microenvironment.

### *Ex-vivo* analysis of the acquired peripheral CD19^+^CD5^+^ population following ibrutinib treatment of MCL patients demonstrates high sensitivity to ABT-199

**Figure 4:**
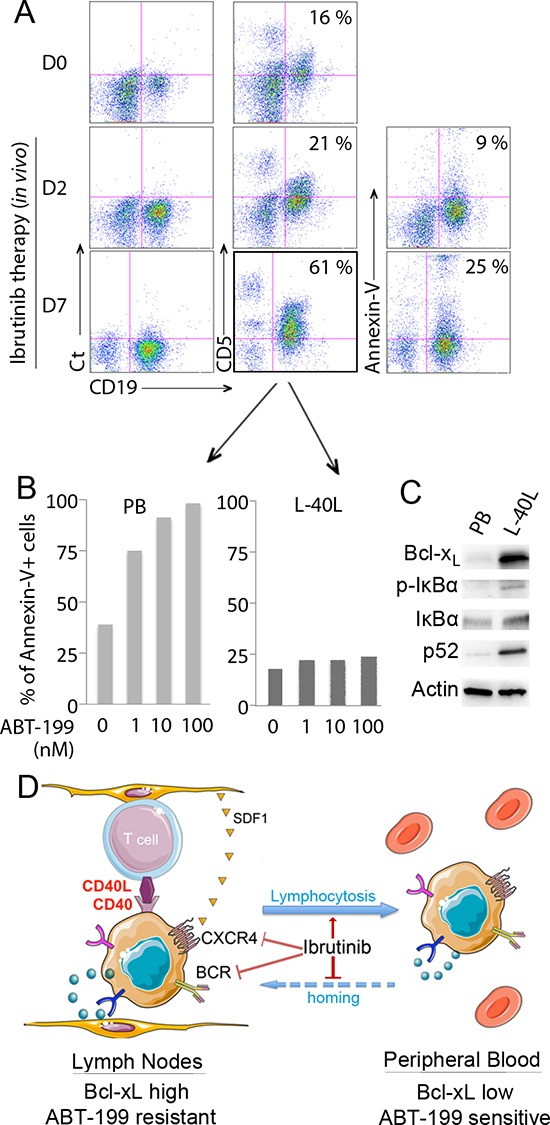
Sensitivity of mobilized primary MCL cells to ABT-199 in a patient treated with ibrutinib **(A)** Peripheral blood (PB) cells from a MCL patient were collected before (D0) and after ibrutinib treatment (D2 and D7) and labeled with CD19-APC and CD5-FITC. The percentage of CD19^+^ CD5^+^ population is indicated in each dot plot. In addition, apoptosis of CD19^+^ MCL cells at D2 and D7 was assessed by Annexin V-FITC staining with the percentage of apoptotic cells indicated in each dot plot. **(B)** After 7 days of *in vivo* ibrutinib single-agent treatment, freshly isolated *de novo* CD19+CD5+ PB cells were cultured with increasing doses of ABT-199 for 24 hours directly or after coculture with L-40L cells. Cell death was assessed by Annexin V-FITC and CD19 staining. **(C)** Expression of Bcl-x_L_ and activation of the classical (p-IkBα, IkBα) or alternative (p52) NF-kB pathway was analyzed in *de novo* CD19+ MCL peripheral blood cells with or without L-40L coculture. **(D)** Schematic representation of ibrutinib mechanism of action in MCL cells. By inhibiting BTK, ibrutinib neutralize both BCR and CXCR4 axis, resulting in egress of MCL cells from the protective microenvironment into peripheral blood.

Ibrutinib is an irreversible BTK inhibitor that displays significant antitumor activity in MCL [21]. By inhibiting BCR and chemokine-mediated stromal adhesion of MCL, ibrutinib has been shown to induce a redistribution of lymph node-resident MCL [22]. Thus, we investigated the lymphocyte population in the peripheral blood of a patient that received ibrutinib (560 mg/d) every day for days 1–7 while treated in the French compassionate use program. Blood was collected and analyzed for the presence of CD19^+^ CD5^+^ MCL cells before treatment and at days 2 and 7 following ibrutinib treatment. As shown in Fig. 4, the peripheral CD19^+^ CD5^+^ lymphocyte population increased from 16% to 21% after 2 days and to 61% after 7 days of ibrutinib treatment, indicating a cellular mobilization (lymphocytosis) from tissues as previously described (Fig. 4A) [22]. Annexin-V staining demonstrated that only 9% or 25% of the tumor cell population undergoes spontaneous apoptosis at days 2 and 7 following ibrutinib treatment respectively (Fig. 4A). In order to analyze the cytotoxic efficiency of ABT-199 on tumor cells mobilized from lymph nodes, peripheral blood population obtained on day 7 was treated with increasing doses of ABT-199 for 24 hours. We found that the CD19^+^ population was highly sensitive to ABT-199, with a LD50 < 1 nM, confirming that tumor cells mobilized from tissues following ibrutinib treatment could be efficiently targeted by ABT-199 (Fig. 4B). The ABT-199 sensitivity of ibrutinib-mobilized cells was further confirmed in a second patient who displayed an increase absolute lymphocyte count (1.9 fold) 10 days after ibrutinib (Supplementary Figure S2A–S2C). *Ex-vivo* co-culture of the peripheral blood population obtained on day 7 on L-40L confirmed ABT-199-resistance (Fig. 4B, Supplementary Fig. S2C) as well as NFkB activation and Bcl-x_L_ up-regulation (Fig. 4C). Thus, we hypothesize that without ibrutinib-specific inhibition of homing into the lymph nodes, circulating cells would become resistant to ABT-199. According to our hypothesize, we demonstrated that while ibrutinib increased the sensitivity of MINO cells to ABT-199, the activity of ibrutinib was not sufficient to significantly reverse the protection observed on L-40L co-culture (Supplementary Fig. S3).

## DISCUSSION

The opportunity to induce apoptosis by selectively targeting Bcl-2 with ABT-199 is a potentially promising therapeutic approach in hematological malignancies [10, 23, 24]. MCL cell lines showed a similar sensitivity to ABT-199 than the one previously described for ABT-737 [18], despite the selective affinity of ABT-199 for Bcl-2 but not Bcl-x_L_. However, ABT-199 efficiency may be increased thanks to a higher affinity to its selective target Bcl-2 (x100 fold) [10]. Indeed, we observed that ABT-199 kills 3 out of 8 MCL cell lines while it is broadly active against all primary MCL cells tested (*n* = 11) at low doses (LD_50_ < 10 nM) (Table 1). We next demonstrated that ABT-199 sensitivity is significantly correlated to *BCL2/(MCL1+BCLXL)* ratio indicating that both Mcl-1 and Bcl-x_L_ play a key role in intrinsic ABT-199 resistance (Fig. 1). Of note, until now we didn’t observe intrinsic resistance to ABT-199 in the primary MCL cells tested. However, analysis of a larger number of primary cells could potentially reveal ABT-199 resistance due to specific mutation in the Bcl-2 target or in Bax effector protein as recently reported [25].

Accumulating data indicate that the microenvironment is a critical factor for cancer cell survival and thus has to be considered for the development of successful new treatment strategies. In this respect, special attention has been paid to modifications occurring in CLL cells stimulated by fibroblasts expressing CD40L, resulting in ABT-737 resistance [14, 15, 26]. Using this coculture system to mimic the microenvironment within lymph nodes, we have shown here that CD40 stimulation of MCL cell lines and patient samples significantly reduce ABT-199 sensitivity (Fig. 2). We demonstrated that (i) peripheral blood MCL cells express low level of Bcl-x_L_ and are highly sensitive to ABT-199, (ii) upon CD40 stimulation there is an increase in Bcl-x_L_ protein level but not Mcl-1 or Bcl-2, and (iii) that silencing of BCL-x_L_ overcomes ABT-199 resistance induced by CD40 stimulation. Taken together our study highlight a critical role for Bcl-x_L_ in microenvironment-dependent protection of MCL cells to ABT-199.

As previously reported [20], we confirmed that NF-κB-signaling pathway is involved in CD40-dependent Bcl-x_L_ up-regulation (Fig. 3). Mechanistically, our results imply that over-expression of Bcl-x_L_ alters the balance between pro-and anti-apoptotic Bcl-2 family proteins and it is the tempting to hypothesize that Bcl-x_L_ is able to capture the pro-apoptotic proteins endogenously bound to Bcl-2 and released under ABT-199 treatment. Of note, among the three MCL cell lines highly sensitive to ABT-199, MINO cells lacks Bim expression in contrast to MAVER-1 and GRANTA-519 cells, indicating that the efficacy of ABT-199 is not affected by the absence of Bim (data not shown).

It was shown recently that ibrutinib, a highly potent oral Bruton's tyrosine kinase (BTK) inhibitor, interferes with the homing of MCL cells into secondary lymphoid organs and/or bone marrow through the inhibition of chemokine and BCR signaling [22]. In agreement with this mechanism of action, ibrutinib induces a concomitant increase of circulating MCL cells [22]. Since circulating primary MCL cells are highly sensitive to ABT-199, it would suggest that combining ABT-199 with ibrutinib could be a very effective therapy for MCL patients. Indeed, we have demonstrated that ABT-199 resistance is rapidly lost when MCL cells detach from CD40L expressing fibroblast cells and that peripheral MCL cells isolated from a patient undergoing ibrutinib treatment were sensitized to ABT-199-mediated killing. Taken together, these results indicate that ABT-199 resistance can be overcome when MCL cells escape from the microenvironments (Fig. 4D).

Based on high response rates in a phase 2 clinical data, ibrutinib was recently approved by the FDA for the treatment of MCL [21]. However, approximately one third of patients did not respond and some became resistant to ibrutinib during treatment [21, 27, 28]. Because ABT-199 kills MCL cells through a distinct mechanism of action and is particularly potent against peripheral MCL cells mobilized by ibrutinib, these agents could be highly complementary and beneficial to patients with significant unmet medical need. Their favorable toxicity profiles may also facilitate their combination in future clinical trials. Furthermore, a recent study demonstrated synergistic effect of the ibrutinib and ABT-199 combination on apoptosis induction in several MCL cell lines [29]. However, while ibrutinib increased the sensitivity of MINO cells to ABT-199, this drug combination was not sufficient to reverse the protection induced by the CD40/CD40L interaction. These last results reinforce the interest of sequential strategy taking advantage of ibrutinib-induced lymphocytosis.

In conclusion, the Bcl-2-selective BH3 mimetic ABT-199 is a promising agent for the treatment of B-cell malignancies including MCL and may be especially attractive in combination with BCR signaling inhibitory drugs such as ibrutinib, which can drive malignant cells out of the protective microenvironment of lymph nodes and bone marrow.

## MATERIALS AND METHODS

### MCL cells and cell lines

MCL cell lines JeKo-1, MINO, REC-1, MAVER-1 were purchased from DSMZ (Braunschweig, Germany), Z138 was purchased from ATCC (Manassas, USA), GRANTA-519 and UPN-1 were kindly provided by Dr. V. Ribrag (Institut Gustave Roussy, Villejuif, France) and Dr B. Sola (IFR 146, University of Caen, France), respectively. Cell lines were maintained in RPMI-1640 medium supplemented with 10% FCS and 2 mM glutamine. Primary cells were obtained after informed consent from MCL patients treated at the department of clinical hematology from the University hospital of Nantes, France. The patient described in Fig. 4 received ibrutinib single agent therapy (560 mg/d) and achieved a partial response lasting 4 months before progression. The patient described in Supplementary Fig. S2 was still under ibrutinib single agent therapy (560 mg/d) at the time of publication. Peripheral MCL cells from blood were purified after Ficoll-Hypaque separation with immuno-magnetic anti-CD19 beads when MCL infiltration was less than 90% (Miltenyi, Paris, France). Primary MCL cells were cultured in RPMI-1640 supplemented with 10% FCS and 2 mM glutamine.

Parental or CD40L-expressing mouse fibroblast L cells were kindly provided by Dr T. Defrance (Lyon, France). L cells were cultured in RPMI-1640 supplemented with 10% FCS and 2 mM glutamine. For the co-culture experiments, L cell were irradiated with 35 Gray or treated with mitomycin and seeded (2.5 × 10^4^ cells/mL) 6 to 24 hours before MCL cell lines (2.5 × 10^5^ cells/mL) or primary cells (5 × 10^5^ cells/mL). ABT-199 was then added for 48 hours.

### Antibodies and reagents

The following antibodies were used for flow cytometry analysis: anti-Apo2.7-PE, anti-CD5-FITC, anti-CD19-APC and control IgG1-FITC mAbs were purchased from BD Biosciences (Le Pont de Claix, France). Analysis of protein expression was conducted by immunoblotting using the following primary antibodies: anti-Bcl-2, anti-IκBα and anti-phosho-IκBα (Cell Signaling, Saint Quentin en Yvelines, France), Anti-Mcl-1 (S19) (Santa Cruz Biotechnology, Santa Cruz, CA), anti-Bcl-x_L_ (BD Biosciences, Le Pont de Claix, France), Anti-NF-κB p52 Antibody and anti-actin (Merck Millipore, Lyon, France). ABT-199 was kindly provided by Abbvie Laboratories (North Chicago, IL, USA) and the selective BTK inhibitor ibrutinib (PCI-32765) was obtained from Selleck Chemicals (Souffelweyersheim, France).

### Viability assays

Cell death in MCL cell lines was assessed by using Apo-2.7 staining (BD Biosciences Le Pont de Claix, France). Cell death in CD19^+^ primary MCL cells was assessed by Apo-2.7 staining combined with an analysis of altered cellular morphology (lower FSC). Alternatively, apoptosis of primary cells was assessed by Annexin V-FITC staining (Beckman coulter). Fluorescence was analyzed on FACSCalibur (Cytocell, SFR Bonamy).

### siRNA transient transfections

Control non-targeted small interfering siRNA (si Ct) and siRNA against *BCLXL* and *MCL1* were purchased from Thermo Scientific (Courtaboeuf, France). Z138 and MINO cell lines were electroporated using a Nucleofector system (Amaxa, Lonza, Basel, Switzerland) according to the manufacturer's instructions. Cells (5 × 10^5^/ml) were suspended in Nucleofector solution T or SF and electroporated in the presence of 10 μmol/L siRNA (T01 for MINO, CM150 for Z138 and DN100 for JeKo-1). The gene-silencing effect was evaluated by immunoblot analysis.

### Quantitative real-time PCR

Quantitative PCR was performed as previously described [30]. TaqMan gene expression assays for *BCL2* (Hs00608023_m1), *MCL1* (Hs00172036_m1), *BCLXL* (*BCL2L1*; Hs00236329_m1) and RPL37a (Hs01102345_m1) were purchased from Applied Biosystems. The following thermal cycling parameters were used: 50°C for 2 min for optimal AmpErase UNG activity and then 40 cycles at 95°C for 30 s and 60°C for 1 min. Amplification of the housekeeping gene *RPL37a* was conducted for each sample as an endogenous control.

### Immunoblot analysis

Cells were collected and lysed in lysis buffer containing 10 mM Tris, pH 7.6, 150 mM NaCl, 5 mM EDTA and 1% TritonX100, 2 mM PMSF and 2 mg/ml aprotinin. Immunoblot analysis was performed according to published protocols [18].

## SUPPLEMENTARY FIGURES


