# Supplementary material for: Biological rational for sequential targeting of Bruton tyrosine kinase and Bcl-2 to overcome CD40-induced ABT-199 resistance in mantle cell lymphoma
Source: Oncotarget. 2015 Mar 5;6(11):8750–9. doi: 10.18632/oncotarget.3275 (PMC4496181; doi:10.18632/oncotarget.3275)
Supplement: Supplementary file 1 [file oncotarget-06-8750-s001.pdf]

SUPPLEMENTARY FIGURES

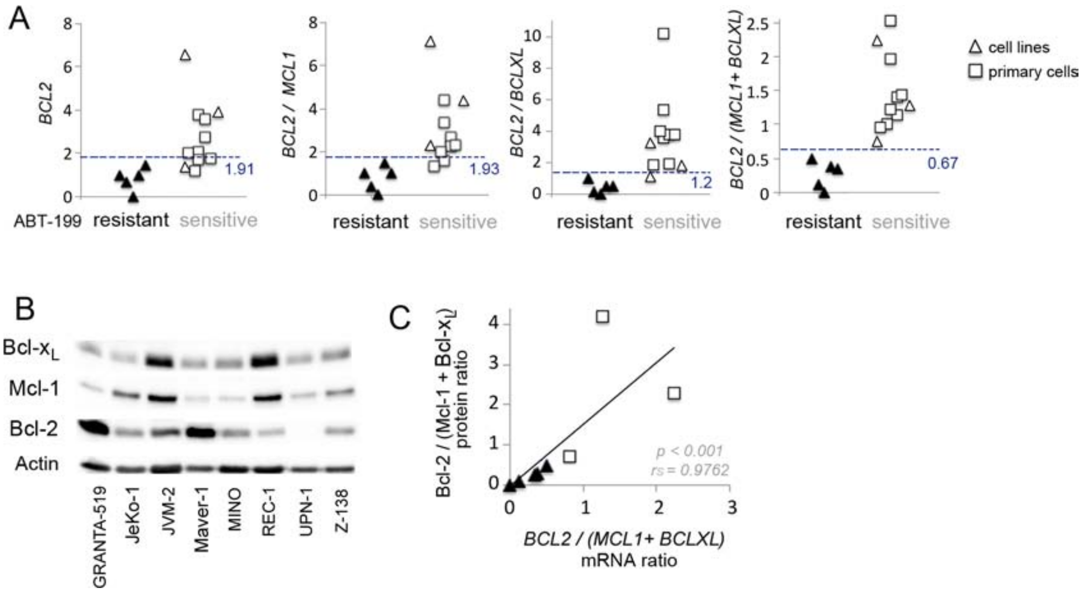

Supplementary Figure S1: MCL cell sensitivity to ABT-199 correlates with the Bcl-2/(Mcl-1+Bcl-xL) ratio. A. Correlative analysis of ABT-199 sensitivity with BCL2 mRNA expression as well as *BCL2/MCL1, BCL2/BCLXL* and *BCL2/*(*MCL1+BCLXL*) mRNA ratio were addressed. Cut-off values are represented by the dot line and were determined as: mean of resistant cells mRNA ratio + (standard deviation) x 2. The true positive rate (sensitivity) was determined by Receiver Operating Characteristic (ROC) curve analysis: *BCL2* 63%, *BCL2/MCL1* 82%, *BCL2/BCLXL* 91%, *BCL2/*(*MCL1+BCLXL*) 100%. **B.** Immunoblotting analysis of Bcl-xL, Mcl-1 and Bcl-2 in the MCL cell lines panel (n = 8). **C.** Bcl-2/(Mcl-1+Bcl-xL) protein ratio was determined after quantification of protein expression shown in B and strongly correlates to the *BCL2/*(*MCL1+BCLXL*) mRNA ratio (spearman, $p < 0.001$).

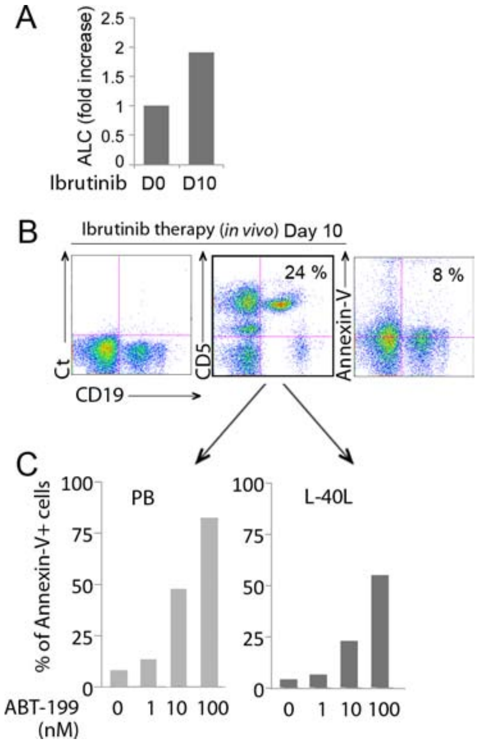

Supplementary Figure S2: Sensitivity of mobilized primary MCL cells to ABT-199 in a patient treated with ibrutinib. A. Increase of Absolute Lymphocyte Count (ALC) is observed 10 days after Ibrutinib treatment (D10) in the peripheral blood of a MCL patient. **B.** Peripheral blood (PB) cells were collected 10 days after Ibrutinib treatment (D10) and labeled with CD19-APC and CD5-FITC. The percentage of the CD19+CD5+ population is indicated. In addition, apoptosis of CD19+ MCL cells at D10 was assessed by Annexin-V-FITC staining. **C.** After 10 days of *in vivo* ibrutinib single-agent treatment, freshly isolated *de novo* CD19+CD5+ PB cells were cultured with increasing doses of ABT-199 for 24 hours directly or after coculture with L-40L cells. Cell death was assessed by Annexin-V and CD19 staining.

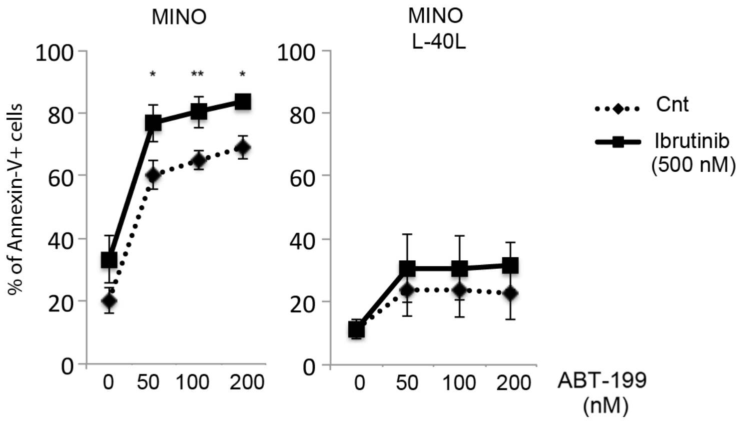

Supplementary Figure S3: *in situ* Ibrutinib and ABT-199 synergy is not sufficient to reverse CD40L-induced resistance. MINO cells were cultured alone or on CD40L-expressing fibroblasts L (L-40L) for 24 hours before being exposed to Ibrutinib and ABT-199 at indicated doses. Cell death was assessed in triplicate by using Annexin-V staining. *p*-value was determined using Student's *t* test: $*p < .05$; $**p < .01$.
